# The Potential Value of Probiotics after Dental Implant Placement

**DOI:** 10.3390/microorganisms11071845

**Published:** 2023-07-20

**Authors:** Jia Xu, Chenfeng Chen, Shuaiqi Gan, Yihan Liao, Ruijie Fu, Chuping Hou, Shuhan Yang, Zheng Zheng, Wenchuan Chen

**Affiliations:** 1 State Key Laboratory of Oral Diseases and National Clinical Research Center for Oral Diseases, West China Hospital of Stomatology, Sichuan University, Chengdu 610041, China; xujiactu@163.com (J.X.); isiss@163.com (C.C.); sqgan@foxmail.com (S.G.); houchuping@hotmail.com (C.H.); zhengzheng942462@163.com (Z.Z.); 2 Department of Oral Prosthodontics, West China Hospital of Stomatology, Sichuan University, Chengdu 610041, China; 3 Department of General Dentistry, West China Hospital of Stomatology, Sichuan University, Chengdu 610041, China; 4 Jinjiang Out-Patient Section, West China Hospital of Stomatology, Sichuan University, Chengdu 610041, China

**Keywords:** probiotics, dental implant, bone regeneration, bone homeostasis, osseointegration, wound healing, peri-implantitis

## Abstract

Dental implantation is currently the optimal solution for tooth loss. However, the health and stability of dental implants have emerged as global public health concerns. Dental implant placement, healing of the surgical site, osseointegration, stability of bone tissues, and prevention of peri-implant diseases are challenges faced in achieving the long-term health and stability of implants. These have been ongoing concerns in the field of oral implantation. Probiotics, as beneficial microorganisms, play a significant role in the body by inhibiting pathogens, promoting bone tissue homeostasis, and facilitating tissue regeneration, modulating immune-inflammatory levels. This review explores the potential of probiotics in addressing post-implantation challenges. We summarize the existing research regarding the importance of probiotics in managing dental implant health and advocate for further research into their potential applications.

## 1. Introduction

Oral health issues are a matter of global concern. Dentition defect and tooth loss have a profound influence on people’s health and quality of life [1]. Since Professor Brånemark introduced the theory of “osseointegration”, the field of oral implantology has experienced vigorous development [2]. Due to its excellent restorative effects on oral function and aesthetics, dental implantation has become a common and popular treatment for dentition defect [3,4].

The success of implant surgery depends not only on the skill of the dentist during the procedure but also on factors such as post-operative wound healing, osseointegration, and alveolar bone repair and regeneration, all of which significantly affect the final outcome of the implant. This is particularly crucial in cases involving multiple teeth or challenging surgical procedures. After the implantation of the implant into the jawbone, the initial process involves the differentiation, migration, and adhesion of osteoblasts to the surface of the implant [5,6]. Early formation of immature woven bone occurs. Over the following months, bone remodeling is completed, leading to the formation of dense lamellar bone [6]. Implants and the bone that surrounds them are biologically interconnected by osseointegration, a fundamental process that produces a functional and stable connection between the two. However, implant osseointegration can not be successfully completed by everyone, such as patients with diabetes and osteoporosis [7,8,9,10]. While achieving successful osseointegration and subsequent prosthetic rehabilitation is significant, ensuring the long-term health and stability of the implant-supported restoration remains a significant concern for both clinicians and patients in clinical practice [3]. Bone tissue exhibits dynamic remodeling and is subject to resorption [11] under the influence of various intrinsic and extrinsic factors, including patient-specific characteristics and pathological conditions. Loss of bone around the implant is called marginal bone loss [12,13]. Marginal bone loss not only affects implant stability but can also have a substantial impact on the aesthetic outcome for the patient [13]. Among the factors that compromise implant stability and marginal bone loss, peri-implant diseases and associated inflammatory reactions represent the most prevalent and clinically significant challenges. Similar to the impact of periodontal disease on natural teeth, peri-implantitis poses obstacles to the health of dental implants [14]. The prevalence of peri-implantitis is notably high, reaching up to 43% in Europe, South America, and North America [15]. It is an infectious disease primarily induced by various pathogens within the oral cavity. The peri-implant soft and hard tissues are gradually destroyed by this process [15,16]. Ultimately, this can result in implant loosening and failure [16]. According to the latest insights from osteoimmunology, marginal bone loss around dental implants is not solely attributed to the action of pathogenic bacteria of peri-implant diseases [11]. Immune-mediated inflammatory responses can also influence the peri-implant bone [11], which involves the overall systemic condition of the patient. This further emphasizes the significant role of the immune system in implant dentistry. There are still many challenges that dentists need to solve after implantation surgery. It is crucial to recognize that post-implant treatment requires ongoing maintenance and care by both the dentist and the patient.

The oral cavity, as a complex microbial ecosystem, maintains a close relationship between oral microbiota and the human body [17,18]. Whether oral mucosal wound healing or subsequent peri-implant diseases, it has been demonstrated that local microbiota plays a significant role in these processes. However, not all microorganisms are harmful [19]. Certain types of microorganisms naturally occurring in the human body and the environment possess beneficial functions for the body and hold significant importance in health management. These microorganisms are commonly referred to as “probiotics” [19]. As defined by the Food and Agriculture Organization (FAO) and the World Health Organization (WHO), probiotics are live microorganisms that, when given in adequate amounts, have a beneficial effect on the body [20,21,22]. Typically, probiotics belong to certain strains of bacterial species, for example, *Lacticaseibacillus rhamnosus*, *Lactobacillus acidophilus*, *Lactiplantibacillus plantarum*, *Lacticaseibacillus casei*, *Limosilactobacillus reuteri*, *Lacticaseibacillus paracasei*, *Bifidobacterium longum*, *Bifidobacterium infantis* and *Bifidobacterium animalis* [22,23]. *Bacillus subtilis* is an example of a species within the *Bacillus* genus known for its probiotic properties [23]. In addition, *Akkermansia muciniphila* is now considered a new generation of probiotics that play beneficial roles in various diseases [24]. In addition to the aforementioned probiotics in the gut, within the oral microbiome, *Streptococcus salivarius* stands out as a prominent representative and is widely recognized as a probiotic. It plays a significant role in the prevention of dental caries and periodontal disease [25,26].

Throughout history, the regulatory effects of probiotics on gut microbiota have been acknowledged, suggesting their potential in preventing and managing gastrointestinal disorders [27]. However, the impact of probiotics extends beyond the gastrointestinal tract. Emerging evidence highlights the pivotal role of probiotics in modulating systemic tissues and organs in the human body [28,29,30], particularly in the realm of immune regulation. This phenomenon is exemplified by the widely recognized and extensively studied concepts of the gut–brain axis and the gut–bone axis, which elucidate the intricate interplay between the gut and other physiological systems at a molecular and cellular level. Recent abundant high-quality studies have revealed the significant role of probiotics in the prevention and treatment of tumors by modulating the immune system’s functionality and utilizing their own byproducts [31]. Bender et al. discovered that *Lacticaseibacillus rhamnosus* enhances the production of IFN by CD8+ T cells in the vicinity of melanoma, thus effectively augmenting the immune-mediated anti-tumor response [32]. In the context of the lungs, oral administration of probiotics has been shown to regulate pulmonary immune function, thereby reducing the incidence and severity of respiratory allergic diseases and acute respiratory infections [22,33]. Sun et al. suggest that probiotics have potential as a therapeutic approach in the occurrence and progression of type II diabetes [34,35]. Notably, Nole et al. summarized the connection between gut probiotics and the skin, highlighting the beneficial effects of probiotics in modulating immune system function and maintaining epithelial barrier integrity, thus offering potential benefits for conditions such as atopic dermatitis, acne, and wound healing [36]. Moreover, emerging evidence indicates that probiotics also exert positive influences on cognition, emotions, and other aspects of human behavior [37,38].

The oral cavity, as part of the digestive system, is susceptible to the potential benefits of probiotics. Probiotics are commonly used to influence oral diseases [39]. These probiotics have been found to antagonize pathogens and inhibit their virulence, thus playing a positive role in maintaining oral health and preventing diseases. Numerous studies have applied probiotics in the treatment of oral mucosal diseases, periodontal disease, dental caries, peri-implant diseases, and even oral cancer, with specific administration recommendations provided [39,40,41,42,43,44]. It is crucial to recognize that post-implant care in the field of oral implantology requires not only local effects but also systemic preparations. The subsequent health of dental implants involves aspects such as soft tissue healing, bone tissue regeneration, control of pathogenic bacteria around implants, and modulation of immune system-mediated inflammatory responses. Considering the significant roles that probiotics play systemically and locally, it appears plausible that probiotics may further address some issues encountered after dental implant surgery. Harnessing the potential of microorganisms to promote oral implant health seems to be a viable approach. This article aims to summarize the potential roles of probiotics in dental implant health and provide feasible directions for future research.

## 2. The Role of Probiotics in Bone Regeneration and Bone Homeostasis

Successful oral implant restoration relies on stable implants, and the prerequisite for implants to function optimally is stable and healthy bone tissue. Once the implants are placed by the oral surgeon, the relationship between bone and implants becomes interdependent, with their success or failure intertwined. The theory of osseointegration, initially proposed by Professor Brånemark, is still widely accepted and used today [45]. The fundamental requirement for implants to achieve proper functionality is good osseointegration, which serves as the theoretical basis and key to implant success [46]. The classic definition of osseointegration is the direct contact and close binding between the implant and bone tissue, without any other intervening tissue components [5,47]. Some researchers currently consider osseointegration as a foreign body reaction, where the interface generates compact bone as a defensive response, and oral implants utilize this kind of reaction [48]. This emphasizes the influence of the immune system on osseointegration. Regardless of the specific definition, osseointegration requires the ability of osteogenic differentiation and bone regeneration [6]. Due to similarities in osteogenic activity, inflammatory response, and angiogenesis, osseointegration is often compared to processes such as fracture healing, although there are some differences [49]. For patients commonly encountered in clinical practice who have insufficient bone volume or bone defects, the current conventional approach in the field of oral implantology is to perform bone augmentation surgeries, such as guided bone regeneration and maxillary sinus floor lifting [50,51]. These procedures involve the use of osteoconductive or bone-inductive materials prior to or during implant surgery, followed by a period of several months to allow for bone tissue regeneration. The success of these procedures greatly depends on the patient’s ability for bone regeneration [52,53].

It is not only the bone-forming ability and bone quality during the early stages of implant surgery that concern oral implantologists. The quality and quantity of the bone surrounding the implant and the bone augmentation area also require attention after the surgery [54]. Marginal bone loss around implants is a common occurrence, especially in elderly patients and those with underlying diseases [7]. Even in the absence of peri-implant diseases or poorly placed restorations, marginal bone loss can still occur around the implants. Any marginal bone loss that occurs after the initial stages of implantation can potentially affect the longevity of the implant [12]. Therefore, post-implantation stability of the bone volume in the surgical area is also crucial. From both the pre-implantation and post-implantation perspectives, the patient’s bone-forming ability and bone homeostasis are essential factors. Enhancing the patient’s bone regeneration ability and reducing bone resorption are directions that ensure the success of implant treatment.

In recent years, the beneficial effects of probiotics on systemic bone tissue have been gradually discovered, even in the absence of direct contact [55,56]. Generally, the influence of probiotics on bone is considered to be remote, as bone tissue is typically considered a completely sterile environment. However, there is a growing awareness of the significant role of gut microbiota, referred to as the gut microbiota–bone axis, in bone health [57,58]. Within this context, probiotics, as a specific group of gut microbiota, play an important role.

The evidence for the beneficial effects of probiotics initially came from studies on fracture healing and osteoporosis treatment, indicating their systemic effects. Numerous animal and human studies have found that the administration of various probiotic strains, such as *Lactobacillaceae* and *Bifidobacterium* species, can accelerate fracture healing, promote osteogenic differentiation, and improve bone trabecular parameters [59,60,61,62]. It has been suggested in the literature that supplementation with specific strains of *Bifidobacterium*, particularly in the elderly population, can expedite fracture repair [62]. In models of osteoporosis induced by various factors, administration of probiotics such as *Lactobacillaceae* and *Bifidobacterium* has been shown to increase bone density, attenuate bone loss, and maintain bone homeostasis [56,61,63,64,65,66]. In clinical research, the effects of probiotic supplementation have been comparable to those of vitamin D and calcium supplementation, and it has been suggested as one of the therapeutic approaches for osteoporosis patients [67,68]. In healthy individuals, probiotics such as *Lactobacillaceae* and *Bifidobacterium* induce upregulation of bone-related genes and proteins (Runx2, Sp7, Bmp, Osteocalcin, Osteonectin, Osteopontin, Bonesialoprotein, and collagen type I), promote osteogenic differentiation of stem cells, and enhance bone regeneration capacity [69,70,71,72,73,74,75,76]. In the animal model of Zebrafish, probiotics such as *Lactococcus lactis* and *Bacillus subtilis* can also promote osteogenic differentiation and bone formation [73]. For the health of the jawbone, the role of probiotics is also prominent [77]. There have also been studies exploring the role of local application of probiotics in promoting implant osseointegration, which has shown promising results. Tan et al. found that locally loaded inactivated *Lacticaseibacillus casei* biofilm on the implant surface can accelerate osseointegration and enhance its effectiveness by activating TLR signaling pathways in macrophages, secreting osteogenic factors such as oncostatin M, and improving osteogenic differentiation of mesenchymal stem cells [78]. Additionally, researchers have discovered that surface coating of titanium implants with yeast-derived polysaccharides can stimulate bone tissue to secrete osteogenic factors and promote implant osseointegration [79].

**Figure 1:**
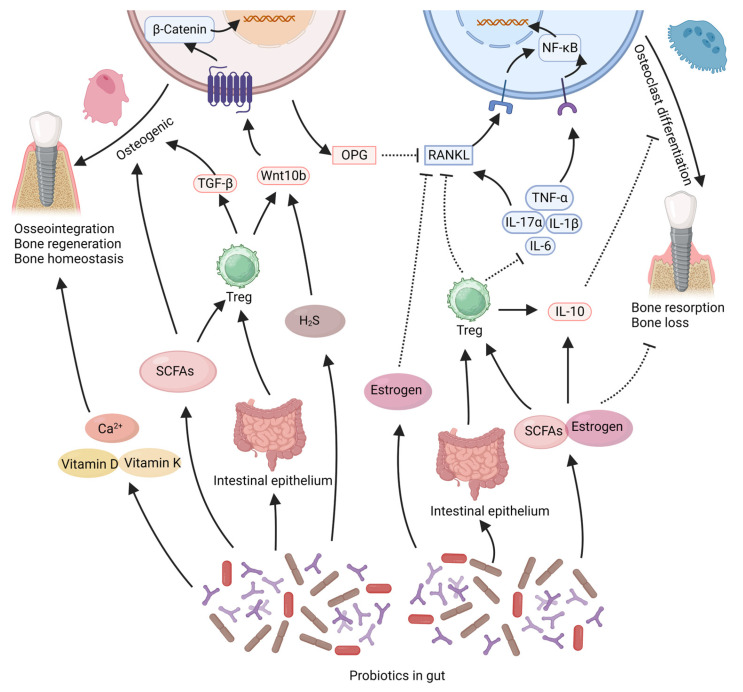
This illustration summarizes the potential beneficial effects of gut probiotics on bone tissue around implants. Probiotics in the intestine can secrete SCFAs, estrogen, which can directly promote osteogenic effects or regulate immunity to maintain bone homeostasis. Probiotics can regulate gut microbiota to enhance the stability of the intestinal epithelium. This can promote the stability of bone tissue mediated by the immune system. In addition probiotics can promote the absorption of nutrients that contribute to bone integration, such as calcium, vitamin D, and vitamin K. The solid arrows in the figure represent “promotion”, while the dashed arrows indicate “inhibition”. SCFAs: short-chain fatty acids, Treg: T regulatory cell. Created with BioRender.com (accessed on 29 June 2023).

Currently, the impact of probiotics on bone tissue primarily originates from the gut. Probiotic strains from gut play a positive role in promoting jawbone health and stability. The mechanisms by which probiotics exert their effects on bone tissue are believed to involve several interconnected aspects (Figure 1).

### 2.1. Probiotics Exert Effects by Products

One distinctive way in which probiotics exert their effects is through the production of beneficial bacterial metabolites, such as short-chain fatty acids (SCFAs), estrogen-like compounds, and vitamins. It has been reported that *Lactobacillaceae* species can produce SCFAs and hydrogen sulfide (H2S) [80], while *Bifidobacterium* species can secrete SCFAs and estrogen-like compounds [81]. *Lactobacillus acidophilus* and Some Bacillus such as *Bacillus subtilis* can also produce vitamin K2, which is considered an important nutrient for bone formation [82]. These factors are widely recognized as crucial contributors to the pro-osteogenic and bone-stabilizing effects of probiotics [69,80,81,83]. In both in vitro and in vivo studies, SCFAs produced by probiotics (such as acetate, propionate, or butyrate) have been shown to increase the production of osteocalcin and osteoprotegerin, stimulate osteogenic differentiation of stem cells, enhance bone formation, and significantly improve trabecular bone parameters [70,84]. SCFAs can also inhibit osteoclast differentiation and suppress bone resorption in mice [85], thus contributing to maintaining bone mass stability. H2S produced by *Lactobacillaceae* species enhances osteoblast activity and prevents osteoblast apoptosis by activating the Wnt signaling pathway in osteoblasts [86]. Estrogen is well-known for its protective effects on bone tissue, and estrogen deficiency leads to bone loss. Probiotics can produce estrogen-like compounds [87], such as equol produced by *Bifidobacterium* species [81,87]. These estrogen-like compounds generated by probiotics can inhibit osteoclastogenesis via the suppression of the RANKL pathway, thereby reducing bone resorption [88]. Additionally, the estrogen-like compounds and SCFAs produced by probiotics are believed to influence systemic immunity and inflammation, which will be further discussed in subsequent sections.

### 2.2. Probiotics Exert Effects by Regulating Inflammation Levels

One important way in which probiotics exert their effects remotely within the gut is by regulating immunity and inflammatory responses. It is increasingly recognized that both implant osseointegration and stability of peri-implant bone tissue are closely associated with immune modulation [12,89]. Immune cells and inflammatory factors greatly influence bone tissue regeneration and stability. Probiotics may regulate systemic and local inflammation levels, thereby promoting bone regeneration and maintaining bone homeostasis [30,62]. The RANKL/RANK pathway, the TNF-α/NF-κB signaling pathway and the Wnt pathway are crucial for regulating osteogenic and osteoclastic activities within bone tissue, and their modulation can be influenced by the inflammatory milieu [90,91,92,93,94]. Reports indicate that *Bifidobacterium longum*, *Lacticaseibacillus paracasei*, *Limosilactobacillus reuteri*, *Lactiplantibacillus plantarum*, *Lacticaseibacillus rhamnosus*, and *Lactobacillus acidophilus* can reduce the expression of the pro-inflammatory cytokine TNF-α within bone tissue, thereby decreasing the activation of the TNF-α/NF-κB signaling pathway, reducing osteoclast activation, and preventing bone loss [81,83,88,95,96,97]. The reduction in TNF-α within bone tissue also leads to decreased RANKL expression, increased osteoprotegerin (OPG) expression by osteoblasts, ensuring osteoblast activity, and reducing osteoclast differentiation [56,88,96,98,99]. *Lacticaseibacillus rhamnosus* and *Limosilactobacillus reuteri* can upregulate the expression of Wnt10b within tissues, activate the Wnt/β-catenin signaling pathway, counteract the inhibitory effects of TNF-α, and enhance osteogenic capacity in mice [70,95,100]. Probiotics such as *Bifidobacterium longum*, *Lacticaseibacillus paracasei*, *Lacticaseibacillus casei*, *Limosilactobacillus reuteri*, and *Lactiplantibacillus plantarum* can also reduce the expression of IL-1β, IL-6, IL-17 within bone tissue while increasing IL-10 expression [83,101,102], thereby inhibiting the osteoclastic effects mediated by the RANKL/RANK and TNF-α/NF-κB pathways, and maintaining stability in the peri-implant bone tissue [81,102,103,104,105]. The balance between Treg cells and Th17 cells plays a crucial role in bone homeostasis [104,106]. Probiotics such as *Lactobacillus acidophilus*, *Lacticaseibacillus rhamnosus* and *Bifidobacterium longum* can upregulate the Treg/Th17 ratio within bone tissue, thereby enhancing osteogenic activity and suppressing osteoclast differentiation [97,103,107,108]. An increase in Treg cell within bone tissue also leads to reduced expression of TNF-α and RANKL, as well as increased expression of IL-10 and Wnt10b [70,100,103].

The ability of probiotics to suppress inflammation may be linked to their capacity to enhance the stability of the intestinal epithelium [62]. The stability of the intestinal epithelium has a direct impact on the translocation of intestinal pathogens, which in turn affects the systemic inflammatory response and the stability of bone tissue [69]. Certain probiotic strains, such as *Bifidobacterium* adolescentis, *Bifidobacterium longum*, *Limosilactobacillus reuteri* and Akkermansia muciniphila have shown the ability to upregulate the expression of the Ocln gene in the intestinal epithelium. This leads to the reinforcement of tight intercellular connections among epithelial cells, resulting in a reduction in systemic inflammation [55,58,59,62,68,109]. These effects play a constructive role in promoting bone regeneration and maintaining bone mass stability [55,58,59,62,68,109]. The ability of probiotics to counteract systemic inflammation may also be linked to their production of compounds such as estrogen-like compounds and SCFAs. These substances have the potential to reduce the levels of pro-inflammatory cytokines (TNF-α, IL-1, IL-6) while enhancing the expression of the anti-inflammatory mediator IL-10 [88,110,111]. Furthermore, these short-chain fatty acids have been implicated in stimulating the migration of Tregs from the intestinal lining to the bone marrow, leading to an increase in the expression of Wnt10b in the bone marrow and facilitating the process of bone regeneration [81,100,112].

### 2.3. Probiotics Exert Effects by Promoting Angiogenesis

In the context of implant osseointegration and bone augmentation surgeries, the effective utilization of osteogenic capacity also relies on the promotion of tissue angiogenesis, which is indispensable [113]. Limited in vivo and in vitro studies have suggested that certain probiotics, whether in live form or in bacterial culture supernatant, can locally stimulate angiogenesis. They have been shown to induce the production of VEGF, thereby promoting endothelial cell growth and migration [114,115,116]. Liu et al. found that in a mouse fracture model, the use of Akkermansia muciniphila in the intestine can also induce type H vessel formation in callus tissue, thereby promoting bone regeneration [109]. However, the current evidence mostly suggests that the angiogenic effects are dependent on the local action of probiotics, and further study is needed regarding their application in the regeneration of peri-implant bone tissue.

### 2.4. Probiotics Exert Effects by Promoting Nutrient Absorption

In addition, probiotics that promote the digestion and absorption of nutrients can also contribute to the health of the skeletal system [55]. Various probiotics, such as *Lactobacillus acidophilus*, *Lacticaseibacillus casei*, *Limosilactobacillus reuteri* and *Bifidobacterium longum* have been shown to enhance the absorption of calcium, vitamin D, and vitamin K [64,117,118,119]. These nutrients are considered crucial for implant osseointegration and stability of the surrounding bone tissue [120,121,122,123,124].

Based on current research findings, the use of probiotics appears beneficial for meeting the skeletal demands of implant treatments. They promote osteogenesis, maintain bone homeostasis, and may even facilitate blood vessel formation.

## 3. The Role of Probiotics in Wound Healing

The speed and quality of wound healing after implant surgery are crucial and easily observable factors. Good wound healing is one of the prerequisites for subsequent osseointegration of the implant and infection control [3,4]. In particular, for implant patients who have undergone bone augmentation procedures, optimal wound healing is a key factor for surgical success [4,113,125]. If the surgical site is extensive, there are higher demands on wound healing [4,113,125]. Some studies suggest that the quality of the mucosa can even affect the occurrence of peri-implantitis [4,126]. Wound healing not only alleviates patient discomfort but also reduces the risk of surgical failure. Furthermore, patients with certain underlying diseases, such as diabetes, experience slower wound healing. Enhancing the speed and quality of wound healing holds significant importance for both patient experience and implant efficacy.

The mechanisms underlying mucosal wound healing are similar to those of the skin; however, due to structural differences, there are subtle variations in the healing process. In contrast to the skin, mucosal wounds exhibit accelerated healing with reduced scar formation [127]. Following the occurrence of an oral mucosal wound, the healing process unfolds through distinct stages: hemostasis, inflammatory response, proliferation, and remodeling. Within minutes of the wound initiation, a cascade of hemostatic reactions is triggered. Subsequently, an inflammatory response ensues over the course of several days [128]. At this stage, neutrophil debridement and macrophage mediated secretion of inflammatory cytokines. Within approximately one week, the inflammatory response subsides, giving way to the proliferation stage, during which fibroblasts proliferate, migrate, and orchestrate collagen deposition and neovascularization, thereby facilitating wound closure. In the subsequent weeks, tissue remodeling occurs, culminating in the further maturation of the dense collagen network [127]. Wound healing is intricately connected with tissue regeneration, infection control, immune modulation, and inflammation regulation. Facilitating epithelialization and modulating excessive inflammatory responses are fundamental strategies to promote optimal quality and expeditious healing of mucosal wounds [127].

**Figure 2:**
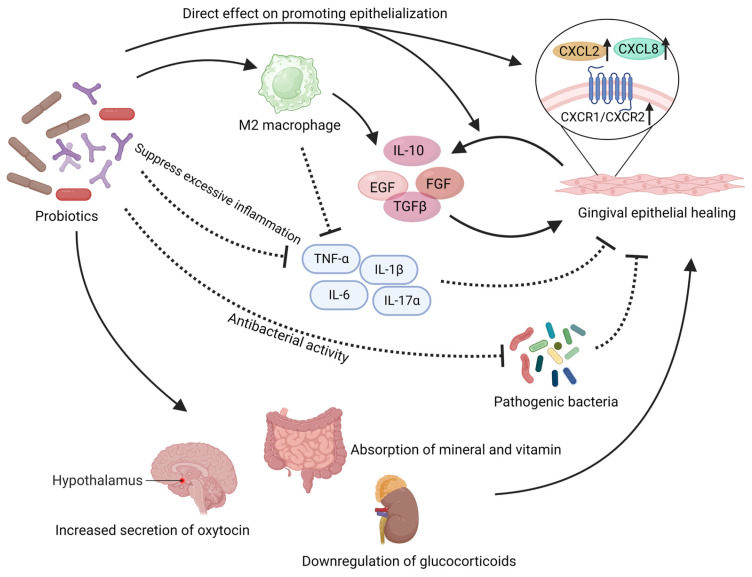
This illustration summarizes the beneficial effects that probiotics may exert in the local mucosal wound. Probiotics have demonstrated their ability to directly promote the proliferation and migration of gingival epithelium both in vivo and in vitro. They can also regulate the level of inflammation in the wound area to accelerate epithelial healing. Probiotics can exert adverse effects on pathogenic microorganisms in the wound area. In addition, probiotics can also regulate the body’s endocrine levels and provide a positive impact on wound healing. The solid arrows in the figure represent “promotion”, while the dashed arrows indicate “inhibition”. EGF: epidermal growth factor, FGF: fibroblast growth factor, TGFβ: transforming growth factor-beta. Created with BioRender.com (accessed on 29 June 2023).

The role of probiotics in promoting wound healing has been increasingly recognized [116,129]. Initially, probiotics were found to contribute to the healing of skin wounds. Local or systemic application of probiotics in patients after burns or surgery can accelerate wound healing and reduce wound related complications [130,131,132]. In particular for patients with wound healing disorders such as diabetes, probiotics can have a good effect on wound healing [133]. Numerous studies focusing on oral mucosal wounds have also revealed the significance of probiotics in oral wound healing. Han et al. demonstrated that local application of *Limosilactobacillus reuteri* lysate significantly enhanced wound healing in a mouse gingival wound model [134]. They also demonstrated the beneficial effects of *Limosilactobacillus reuteri* on wound healing in mouse palatal wounds [135]. Probiotics have the following effects in promoting wound healing (Figure 2).

### 3.1. Probiotics Have Direct Effects on the Gingival Epithelium

The regeneration and migration functions of epithelial cells, fibroblasts, and mesenchymal stem cells are essential for mucosal wound healing and epithelial formation [136]. Certain probiotics have been found to directly enhance cell regeneration and migration capabilities [116]. In in vitro experiments, *Limosilactobacillus reuteri* lysates was shown to activate the potential of gingival mesenchymal stem cells (GMSCs), enhancing their migration capability and expediting the wound healing process [134,135]. Probiotic mixtures comprising various Lactobacillus and Lactococcus have been shown to enhance the growth and migration abilities of human fibroblasts [137]. Studies have found that the lysates of *Lactobacillus salivarius*, *Lactiplantibacillus plantarum* and *Lacticaseibacillus rhamnosus* promote the growth and migration of keratinocytes [138]. Emmanuel et al. demonstrated that *Lactobacillaceae* and *Bifidobacterium* directly enhance migration and proliferation of gingival epithelium in co-culture conditions [139]. In vivo experiments have also shown that *Lactobacillus bulgaricus* and *Lactiplantibacillus plantarum* can increase the local population of fibroblasts, thereby accelerating wound healing [140]. The direct effects of probiotics on gingival epithelial regeneration can be explained through the expression of CXCL and its receptors, CXCR1/CXCR2. *Lacticaseibacillus rhamnosus* has been reported to enhance the expression of CXCL2 and CXCR2 in keratinocytes [139]. Under the influence of *Lacticaseibacillus casei* and *Lactobacillus acidophilus*, *Bifidobacterium* pseudolongum, the secretion of CXCL8 by gingival epithelial cells significantly increases, accompanied by a substantial increase in the surface receptors CXCR1/CXCR2 on gingival epithelial cells [141,142,143]. CXCL2 and CXCL8 can activate CXCR1/CXCR2 receptors in a classical manner, stimulating epithelial cell proliferation and migration [139,144].

Probiotics also exert the ability to stimulate the activation of wound repair-associated growth factors [145], thereby expediting the healing process. Yousef Ashoori proposed that probiotic metabolites such as polysaccharides, lactic acid, and acetic acid present in the supernatant of *Limosilactobacillus reuteri* can effectively trigger the local release of growth factors, including epidermal growth factor (EGF), fibroblast growth factor (FGF), and transforming growth factor-beta (TGFβ) [146]. These growth factors play a pivotal role in the intricate process of wound healing [147,148,149,150,151,152]. Notably, both in vitro and in vivo investigations have demonstrated the profound impact of *Limosilactobacillus reuteri* in activating the PI3K/AKT/β-catenin pathway within mesenchymal stem cells, ultimately culminating in enhanced proliferation, differentiation, and wound closure facilitated by upregulated TGFβ1 expression [134]. Furthermore, the utilization of probiotics derived from kefir has shown promising potential in augmenting the expression of TGFβ1 and FGF genes [137]. Local application of *Lactobacillus bulgaricus* and *Lactiplantibacillus plantarum* has been found to effectively heighten the expression of TGFβ1 in wound tissues [140]. Additionally, the administration of a probiotic mixture consisting of eight strains, including *Bifidobacterium* and *Lactobacillaceae*, has demonstrated substantial elevation in mucosal levels of EGF and TGFβ1, significantly enhancing the regenerative capacity and wound healing efficacy [115]. Studies focusing on the intestine have shown that the use of *Lacticaseibacillus rhamnosus* can enhance the expression of EGF, thereby promoting mucosal protection and regeneration [153,154].

### 3.2. Probiotics Exert Effects by Reversing the Impact of Oral Pathogens

Bacterial dysbiosis has been shown to impair wound healing [135]. In an imbalanced oral microbiota environment, the functionality of oral gingival and palatal mesenchymal stem cells (MSCs) is compromised, resulting in a significantly slowed healing process of oral soft tissues [155]. However, probiotics can counteract the detrimental effects of pathogenic bacteria or reverse their unfavorable impact on tissue repair [135]. Han et al. discovered that *Porphyromonas gingivalis*, a periodontal pathogen, slows down wound healing due to the influence of its lipopolysaccharide (LPS). However, when co-administered with *Limosilactobacillus reuteri* the wound healing speed increased and exceeded that of the control group [135]. Even the ultrasound extract of *Limosilactobacillus reuteri* can reverse the adverse effects of *Porphyromonas gingivalis* LPS on the migration of gingival MSCs [135]. Local application of the supernatant derived from Lactobacillus VITSAMJ1, isolated from goat milk, can inhibit the growth of *Staphylococcus aureus* in the wound area and reduce apoptosis in epithelial cells [145].

### 3.3. Probiotics Exert Effects by Regulating Local Inflammation Levels

Probiotics exert an additional significant mechanism in promoting wound healing, which involves the regulation of the inflammatory response [116,146,156,157]. Excessive inflammation at the site of the wound can cause delays in epithelialization and impede the healing process [127]. Probiotics possess the capability to downregulate pro-inflammatory cytokines while upregulating anti-inflammatory factors. Research conducted by Emmanuel et al. revealed that a variety of *Lactobacillaceae* and *Bifidobacterium* strains (such as *Limosilactobacillus reuteri*, *Lacticaseibacillus rhamnosus*, *Lactobacillus acidophilus*, *Lacticaseibacillus casei*, *Bifidobacterium longum*, *Bifidobacterium* animalis, *Bifidobacterium* breve, *Bifidobacterium* pseudolongum, and *Bifidobacterium* bifidum) can effectively downregulate the expression of IL-1β and TNF-α, which are pro-inflammatory cytokines, in damaged gingival epithelial tissue [142]. In the context of diabetic wound healing, the administration of *Lactobacillus bulgaricus* and *Lactiplantibacillus plantarum* has been observed to significantly reduce the levels of IL-1β and TNF-α [140]. Notably, *Lacticaseibacillus casei* has been reported to modestly inhibit the expression of various pro-inflammatory cytokines, such as IL-1β, IL-2, IL-6, IL-12, IL-17α, IFN-γ, and TNF-α [140], thereby contributing to the facilitation of the wound healing process. *Lacticaseibacillus rhamnosus* not only regulates the levels of inflammatory factors but also exerts an inhibitory effect on immune cells (Th1, Th2, and Th17) to control inflammation [158,159]. Additionally, probiotics upregulate local anti-inflammatory factors. *Lactiplantibacillus plantarum* enhances the expression of IL-10 in post-operative mucosal tissues, contributing to a favorable healing environment [160]. Similarly, *Lactobacillus bulgaricus* can increase the local expression of IL-10 and TGFβ1 in the wound area, effectively reducing inflammation levels and supporting the healing process [140]. The administration of *Limosilactobacillus reuteri* induces the upregulation of the anti-inflammatory cytokine IL-10, thereby minimizing tissue damage at the wound edge and further downregulating the anti-inflammatory mediator IL-17A [161]. Consequently, following probiotic or probiotic supernatant treatment, there is a significant decrease in local inflammation levels [140]. Numerous studies suggest that the beneficial effects of probiotics on wound healing are closely associated with their ability to modulate inflammation [116,162]. By resolving inflammation, macrophages transition into an M2 phenotype, which in turn promotes the release of regenerative growth factors such as FGF, EGF, and VEGF [127,163]. This elucidates the importance of attenuating inflammation for facilitating mucosal healing.

### 3.4. Probiotics Provide Indirect Support for Mucosal Healing

In addition to directly influencing local immunity, inflammation, and tissue repair, probiotics can provide indirect support for wound healing. One understandable pathway is that probiotics can improve the absorption of essential nutrients required for wound healing, particularly vitamins, minerals, and cofactors for tissue repair enzymes [116,164]. Research has shown that probiotics can enhance the absorption of inorganic salts, such as calcium ions [165]. Furthermore, *Lactobacillus rossiae* have been shown to produce vitamin B12 that is beneficial to wound healing [166]. *Limosilactobacillus reuteri* and *Lactobacillus acidophilus* can increase the absorption of dietary vitamin D and E [117,167,168,169,170], which is crucial for wound healing.

Certain studies propose that oral administration of probiotics can exert regulatory effects on systemic immunity, inflammation, and tissue repair. Remarkably, the consumption of *Limosilactobacillus reuteri* lysate alone has been demonstrated to enhance endogenous oxytocin levels [171], a pivotal hormone implicated in promoting wound healing [172,173]. Notably, an augmentation of oxytocin-producing cells within the paraventricular nucleus (PVN) of the hypothalamus adjacent to the third ventricle has been observed in mice treated with probiotic lysate [171]. Clinically, probiotic supplementation has exhibited a substantial elevation in local oxytocin levels within oral mucosal wounds [174]. Moreover, investigations suggest that probiotic intake, including *Limosilactobacillus reuteri*, Lactobacillus helveticus, and *Bifidobacterium longum*, can induce a decline in systemic glucocorticoid levels, thus fostering a conducive milieu for wound healing [171,175].

Furthermore, probiotic supplementation may potentially alleviate post-operative pain perception in patients. A clinical study focusing on mucosal wound healing following tooth extraction revealed a significant reduction in swelling sensation among individuals administered oral probiotics [176]. Moreover, patients receiving probiotics reported fewer sleep disturbances, reduced sick leave duration, higher healing score indices, and improved wound healing outcomes [176]. These findings may be associated with factors such as wound healing rate, infection control, among others, warranting further investigation.

## 4. The Role of Probiotics against Peri-Implant Diseases

The most common biological complication in implantology is peri-implantitis, which refers to the inflammation of the tissues surrounding the implant, including both the soft and hard tissues. These tissues provide essential support and protection for the implant [177]. If peri-implant diseases occur, they can significantly affect the functionality and aesthetics of the implant. Peri-implant diseases are classified into “peri-implant mucositis” (involving only soft tissues) and “peri-implantitis” (involving both soft and hard tissues) [178]. Generally, peri-implant mucositis, if left untreated, can progress to peri-implantitis. Peri-implant diseases can cause damage to the peri-implant soft and hard tissues and directly lead to implant failure [179].

Overall, the incidence of peri-implantitis ranges from 1.1% to 85.0% [8]. A survey conducted on patients with implants for over one year found a prevalence of 10.7% for peri-implantitis at the implant level [180]. Within three years after implant placement, the incidence rates of peri-implant mucositis and peri-implantitis were reported as 29.48% and 9.25%, respectively, based on the number of implants [181]. The most recent epidemiological study conducted in China revealed that after 2.5 years of implantation, the incidence rates of peri-implant mucositis and peri-implantitis were 36.69% and 7.66% at the implant level [182]. Peri-implant diseases pose a significant challenge to the long-term functionality of dental implants and have become a serious public health issue [14].

The primary etiology of peri-implant diseases is microbial infection of the peri-implant tissues [183,184]. Peri-implantitis is characterized by a dysbiosis of the subgingival microbial ecosystem [185]. There are significant similarities between peri-implantitis and periodontitis in terms of etiology and pathological changes. Some studies believe that there is no difference between the pathogens of diseases around implants and Periodontal disease [186]. However, more recent research indicates that the pathogenic bacterial populations in peri-implantitis may have slight differences [185,187,188,189], and there is considerable variation in the microbial profiles among individuals [187]. In addition to the bacterial complexes commonly found in periodontitis, such as Red-Complex Bacteria and other bacteria (including *Porphyromonas gingivalis*, *Fusobacterium nucleatum*, *Prevotella intermedia*, *Tannerella forsythia*, and *Treponema denticola*), peri-implant diseases also frequently involve bacteria such as *Staphylococcus aureus*, *Prevotella nigrescens*, and *Campylobacter* species [177,190]. However, it should be noted that the traditional periodontal pathogens, including *Porphyromonas gingivalis*, *Fusobacterium nucleatum*, *Prevotella intermedia*, *Tannerella forsythia* and *Treponema denticola*, are highly prevalent and play an important role in peri-implant diseases [177,187,188,190,191]. In particular, *Porphyromonas gingivalis*, *Fusobacterium nucleatum*, and *Tannerella forsythia* exhibit high abundance in peri-implant diseases [188]. Under the attack of pathogenic bacteria, the host’s excessive inflammatory response leads to the destruction of peri-implant soft and hard tissues, ultimately resulting in the failure of implant treatment.

**Figure 3:**
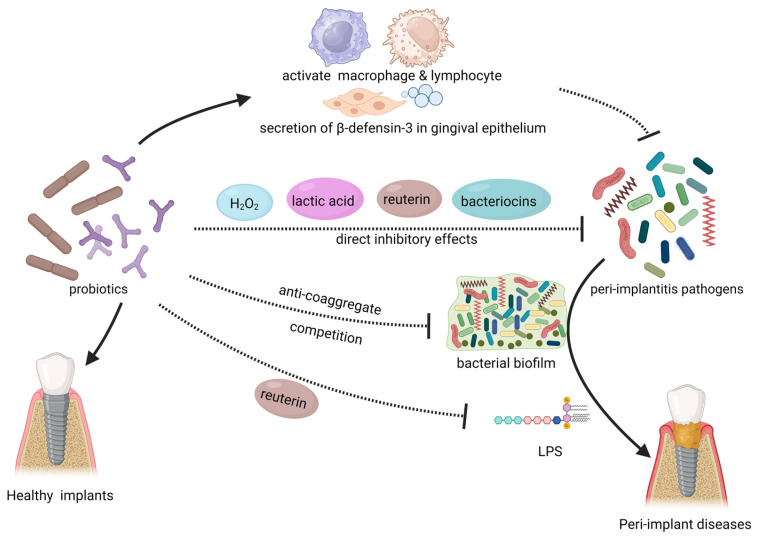
This illustration summarizes the local mechanism of probiotics in combating peri-implant diseases. Probiotics have an inhibitory effect on peri-implantitis pathogens and biofilm in the oral cavity directly. Probiotics can also enhance the body’s immune system and enhance the epithelial barrier. The use of probiotics can also resist the toxic effects of pathogenic bacteria. The solid arrows in the figure represent “promotion”, while the dashed arrows indicate “inhibition”. LPS: lipopolysaccharide. Created with BioRender.com (accessed on 29 June 2023).

Probiotics have long been proven to be a treatment for infections, including dysbiosis and infectious diseases within the oral cavity [192]. They also appear to have targeted benefits for peri-implant diseases. Probiotics play a significant role in regulating the dysbiosis of microbial populations around dental implants. They can effectively inhibit the colonization of pathogenic bacteria in peri-implant tissues, balance the oral microbiota, and thus exert an anti-infective effect [135]. Microscopically, probiotics mainly exert their effects through the following ways (Figure 3).

### 4.1. Direct Inhibitory Effect on Pathogens

Probiotics directly hinder the growth and proliferation of pathogenic microorganisms in the human body. Probiotic strains such as *Weissella cibaria*, *Limosilactobacillus reuteri*, and *Lactobacillus salivarius* have been shown to significantly inhibit the growth of peri-implantitis pathogens such as *Porphyromonas gingivalis*, *Fusobacterium nucleatum*, *Prevotella intermedia*, *Tannerella forsythia*, and *Staphylococcus aureus* in vitro [193,194,195]. They also affect the colonization of these bacteria on titanium surfaces [195]. Vacca et al. demonstrated that *Streptococcus salivarius*, a member of the oral micro-biome, exhibits the ability to inhibit the formation of pathogenic biofilms on implant materials in vitro. These findings suggest that *Streptococcus salivarius* could be utilized for the prevention or treatment of peri-implant diseases [196]. *Bifidobacterium* species exhibit inhibitory effects against various peri-implant disease pathogens, including *Porphyromonas gingivalis* and *Fusobacterium nucleatum*, with particularly strong inhibition against *P. gingivalis* [197,198,199]. The inhibitory effects of probiotics on pathogenic microorganisms may arise from the production of specific substances. Various *Lactobacillaceae* and *Bifidobacterium*, including *Limosilactobacillus fermentum* and *Lactobacillus acidophilus*, exert direct inhibitory effects on peri-implant disease pathogens through producing hydrogen peroxide, bacteriocins and organic acids [200,201,202]. Reuterin (3-hydroxypropionaldehyde) is an antimicrobial metabolite produced by Lactobacillus species. *Limosilactobacillus reuteri*, in previous studies, was found to exert antibacterial effects by utilizing reuterin to oxidize thiol groups in peri-implantitis pathogens [116]. Radaic et al. discovered that *Lactococcus lactis* producing nisin (a bacteriocin) exhibited inhibitory effects against various peri-implantitis pathogens such as *Fusobacterium nucleatum*, *Porphyromonas gingivalis*, and *Treponema denticola* [203]. This particular *Lactococcus lactis* can disrupt the biofilm formation of pathogenic bacteria on the surface of implant materials [203]. As one of the bacteria capable of colonizing the oral cavity, probiotics compete with other oral microbiota, particularly for nutrients and binding sites. Due to its own competitive advantage in terms of nutrients and growth factors, *Bifidobacterium* can competitively inhibit peri-implant disease pathogens such as *Porphyromonas gingivalis* [198,204]. *Bifidobacterium* may have potential implications for bacterial aggregation and biofilm formation of pathogens, as it can compete for binding sites and consequently affect pathogen coaggregation [205,206,207].

### 4.2. Probiotics Prevent Tissue Damage Mediated by Pathogenic Bacteria

Furthermore, certain probiotic strains have the ability to neutralize toxic substances produced by pathogenic bacteria or alleviate the detrimental effects mediated by these pathogens. Han et al. discovered that reuterin, a unique and effective compound secreted by *Limosilactobacillus reuteri*, can mitigate the toxic effects of *Porphyromonas gingivalis* lipopolysaccharide (LPS) on the host [135]. Probiotics may play a role in modulating inflammation locally in the periodontal tissues, thereby alleviating tissue damage and disease progression caused by pathogen-induced inflammation. Flichy-Fernández et al. found that after treatment with *Limosilactobacillus reuteri* in patients with peri-implant mucositis, there was a significant decrease in inflammatory mediators such as IL-1β and TNF-α in peri-implant gingival crevicular fluid [105]. The authors suggested that the local inhibition of excessive inflammatory responses by probiotics is an important factor in relieving clinical symptoms, preventing further tissue destruction, and promoting tissue repair. As mentioned earlier, probiotics have been shown to modulate systemic inflammation in the gut, and this may also have significant implications for mitigating inflammation in the peri-implant region.

### 4.3. Probiotics Enhance Immune Function

Some researchers have found that the direct intervention of probiotics on pathogenic bacteria cannot fully explain their significance in peri-implant diseases [208]. Probiotics can also enhance the host oral mucosa’s resistance to pathogenic bacteria in the peri-implant region [206]. The local upregulation of β-defensins, which are antimicrobial peptides, by probiotics has been widely reported and can help maintain the body’s barriers [116,209]. Invernic et al. found that treatment with *Bifidobacterium* animalis subsp lactis HN019 led to increased secretion of β-defensin-3 in gingival epithelium, thereby enhancing the resistance against pathogenic bacteria such as *Porphyromonas gingivalis* [197]. Moreover, local expression of Toll-like receptor 4 (TLR4) and CD-4, CD-57-positive immune cells was upregulated. These cells play a role in pathogen recognition and activation of innate immunity. The local use of probiotics significantly improves the body’s own defense capabilities [197]. It has also been reported that probiotics possess immunostimulatory extracellular polysaccharides that can activate macrophages and lymphocytes, thereby enhancing immune function [210].

### 4.4. Clinical Studies on the Treatment of Peri-Implant Diseases with Probiotics

Introducing probiotics into the unique environment of the oral cavity holds promising prospects for combating peri-implant diseases. In recent years, researchers have proposed the use of probiotics for the treatment or adjunctive treatment of peri-implantitis. Their main basis is the significant changes observed in clinical indicators of peri-implant diseases in clinical trials or animal studies. Clinical studies have compared the effects of mechanical plaque removal alone or in combination with local application of *Limosilactobacillus reuteri* in the treatment of peri-implant diseases. The results showed that patients who received probiotic supplementation showed better improvements in clinical parameters such as probing bleeding and probing depth, regardless of the extent of the disease [208,211]. Butera et al. suggested that the use of *Limosilactobacillus reuteri*, Lactobacillus brevis, or *Lactiplantibacillus plantarum* in the treatment of peri-implant diseases led to varying degrees of improvement in clinical indices such as bleeding on probing, attachment loss, and plaque index [212]. The latest systematic review indicates that probiotics, as an adjunctive treatment for peri-implant mucositis, have good clinical efficacy, surpassing the use of antibiotics [213,214]. The authors suggest that probiotics should be used as an adjunct to mechanical plaque removal. There is also a systematic review that suggests that probiotics have positive significance in improving clinical parameters such as probe depth in peri-implantitis [215]. However, there are also some studies doubt that the use of probiotics can cure patients who have already developed peri-implant inflammation, which remains to be discussed [216,217,218].

Peri-implantitis is currently challenging to eradicate, and its treatment principle involves the removal of biofilm. Current treatment options include mechanical therapy, the use of antibiotics [15,126,219], and even surgical procedures. Once peri-implant diseases progress to the stage of peri-implantitis, the difficulty of treatment increases significantly. Based on existing research, if left untreated in the early stages, peri-implant mucositis tends to rapidly progress [220]. Considering the cost and difficulty of treating peri-implantitis, the best approach is to intervene before the onset of peri-implant diseases or in the early stages of the disease to prevent further progression [126,184]. Although probiotics are controversial for the cure of peri-implantitis, more than one study emphasizes the clinical significance of probiotics in controlling and preventing peri-implant diseases [105,208,213,221,222]. Increasingly, research suggests that probiotics have a beneficial effect on the early stages of peri-implant diseases, especially peri-implant mucositis, which serves as a precursor to peri-implantitis [213]. Probiotics have shown promising therapeutic effects in this regard, indicating their potential for prevention and control of peri-implant diseases. Tan et al. found that the surface loading of inactivated Lactobacillus biofilm on implants exhibited strong resistance against (MRSA) and could be used to prevent the occurrence and progression of peri-implantitis [78]. Zhu et al. demonstrated that when *Bifidobacteria* first colonize the environment, they can strongly inhibit the colonization and biofilm formation of peri-implant pathogens such as *Porphyromonas gingivalis, Fusobacterium nucleatum*, and *Prevotella intermedia* [200]. *Bifidobacterium* are beneficial for the growth of oral microbiota (*Actinomyces naeslundii* and *Streptococcus mitis*) associated with gingival health while inhibiting the proportion of peri-implant pathogens [199,223]. In studies related to periodontitis, it has been found that early intervention with probiotics in a healthy state significantly reduces the severity of subsequent periodontal disease models [224]. Therefore, these findings suggest that the early use of probiotics may have a more significant preventive effect on peri-implantitis.

Probiotics can play a crucial role in preventing peri-implant diseases by inhibiting the growth of pathogens and influencing the formation of biofilm structures. However, Clinical studies investigating the prevention of peri-implant diseases with probiotics are currently lacking on a large scale. According to existing literature, probiotics such as Lactobacillus and *Bifidobacterium* have shown potential in the prevention and control of peri-implant diseases in the oral cavity. Their single or combined use may become important tools in preventing peri-implant diseases.

## 5. Conclusions and Outlook

Probiotics exert extensive and remarkable effects in the human body. They have the ability to directly influence the prognosis of implants by impacting local hard and soft tissues. Probiotics can also indirectly support post-operative healing and osseointegration of implants by affecting systemic conditions. From a localized perspective, probiotics can promote the regeneration of local soft and hard tissues, exhibit antimicrobial properties against pathogenic bacteria, and reduce local inflammatory reactions. From a systemic standpoint, the ingestion of probiotics can regulate systemic immune-inflammatory responses and enhance the absorption of nutrients. Probiotics also possess the capability to remotely assist in bone regeneration and homeostasis, making them particularly valuable for post-implantation health care.

The utilization of probiotics as an intervention measure holds intrinsic value due to its benefits, including a relatively low safety risk and non-invasive mode of intervention. Probiotics are worthy of widespread utilization [22,225,226,227,228]. However, several considerations need to be addressed before their formal implementation in clinical practice.

Probiotics confer benefits to human health, and the utilization of probiotics has a long-standing history. In general, probiotics are associated with minimal adverse effects on the human body [81,192,229]. While adverse effects of probiotics are really rare in clinical settings, as living microorganisms, it may be prudent to exercise caution when employing probiotics in clinical applications [229]. Probiotics have been associated with several potential and theoretical risks [230]. There is a potential risk of probiotics causing systemic infections [231,232]. Actually, the likelihood of probiotics causing systemic infections is generally low in patients without specific diseases or specific interventions [233,234]. Based on current clinical research, there may be a risk of systemic infection associated with the use of probiotics in patients with short bowel syndrome (SBS), heart valve disease, and those requiring central venous catheters [230,233,235]. Probiotics, due to the secretion of substances such as D-lactic, have been reported in some studies to potentially cause gastrointestinal adverse reactions in a subset of patients undergoing intestinal surgery, such as abdominal cramping, nausea, soft stools, flatulence, and taste disturbance [229,236]. However, compared to patients not receiving probiotics, those who received probiotics generally experienced fewer gastrointestinal reactions [235]. Due to the immunomodulatory effects of probiotics, they may also potentially trigger an exaggerated immune response in the body. Some reports have indicated an increased risk of allergic rhinitis, asthma and atopic allergies in certain individuals after the use of probiotics [233]. However, overall, the occurrence of such events is very low [229]. Probiotics, as bacteria, may potentially undergo antibiotic resistance transfer with pathogenic bacteria. Currently, it is widely believed that the probability of antibiotic resistance transfer in probiotics is very low [231,233,236]. However, as required by the European Food Safety Authority, the genetic nature of clinically relevant antibiotic resistance genes must be explained for probiotics [236]. The gene transfer of probiotics is currently being regulated and monitored. In a study published in *Cell* in 2018, it was reported that probiotics may have long-term negative effects on the recovery of the gut microbiota following antibiotic treatment [237]. Although there is no clinical evidence to suggest that this phenomenon may have adverse health impacts [238], the safety concerns associated with probiotics deserve ongoing attention during their usage. Furthermore, additional clinical trial results and experimental models are needed to further refine the guidelines for probiotic use. It is important to use probiotics within a defined range and regulate their use diligently to ensure their optimal clinical benefits can be realized.

The clinical effects of different probiotic strains vary significantly. Even within the same species of traditional probiotics such as *Lactobacillaceae* and *Bifidobacterium*, distinct strains exhibit diverse properties. For instance, it has been reported that *Limosilactobacillus fermentum* has been shown to reduce the vitality of keratinocytes and suppress re-epithelialization [116,139]. Furthermore, not all probiotic strains exert equal effects on processes such as osteogenesis, wound healing, and combating peri-implantitis pathogens. In addition, the acceptance of different probiotic strains may vary among individuals, which can also influence the effectiveness of probiotics across different individuals [239]. Therefore, the selection of probiotics should prioritize strain specificity. Further research is needed to enhance the evidence concerning different strains in various aspects of dental implants. This can aid in identifying the optimal combination of probiotics and even explore the possibility of developing engineered strains through biotechnology, merging the advantages of different probiotic strains.

Based on current findings, further basic and clinical research is needed for the clinical application of probiotics in implantology. While there is substantial evidence supporting their role in combating peri-implant diseases, promoting oral mucosal wound healing, and enhancing jawbone health and stability, studies specifically focused on implant surgery are still lacking. In our opinion, conducting research on oral implantation in conjunction with probiotics is crucial based on the existing evidence. Moreover, research on probiotics may be more important not only for healthy patients (animals), but also for patients with certain underlying diseases and elderly individuals. Current evidence suggests that probiotics exhibit significant potential in mitigating the impact of underlying diseases, particularly in the context of diabetes and osteoporosis [64,67,133]. In the field of oral implantology, conditions such as diabetes and osteoporosis are generally recognized as high risks for implant health [7,9,10]. However, some researchers now argue that underlying diseases may not be absolute contraindications for implant surgery [7]. Therefore, employing probiotic intervention in implant patients with underlying diseases seems to be even more meaningful, as these patients may derive greater benefits compared to healthy individuals.

Probiotics are significant microorganisms in the human microbiota. Due to their safety and efficacy, probiotics can be applied to various diseases. We have summarized the benefits that probiotics can provide in several aspects of post-implantation. Based on this review, we believe that probiotics hold potential for application in promoting soft and hard tissue healing, maintaining bone homeostasis, and combating peri-implant diseases in oral implantation. We call for further research to explore their potential value.

## Data Availability

Not applicable.
